# Anxiety in the Classroom: Only Girls’ Anxiety Is Related to Same-Sex Peers’ Anxiety

**DOI:** 10.3390/ijerph20010084

**Published:** 2022-12-21

**Authors:** Sandrine Charbonneau, Audrey-Ann Journault, Rebecca Cernik, Charlotte Longpré, Nathalie Wan, Charles-Édouard Giguère, Sonia Lupien

**Affiliations:** 1 Centre for Studies on Human Stress, 7331 Hochelaga Street, Montreal, QC H1N 3V2, Canada; 2 Department of Psychology, University of Montréal, Montreal, QC H3T 1J4, Canada; 3 Research Center, Institut Universitaire de Recherche en Santé Mentale de Montréal, 7331 Hochelaga Street, Montreal, QC H1N 3V2, Canada; 4 Department of Psychiatry, University of Montréal, Montreal, QC H3T 1J4, Canada

**Keywords:** state anxiety, trait anxiety, peers, homophily, same-sex peers

## Abstract

Many teens report experiencing anxiety in school, which can negatively impact their well-being. Considering that adolescents tend to adopt the same behaviors as their classmates with whom they spend, on average, 923 h every year, the current exploratory study (1) assessed whether an association exists between a student’s state anxiety score and his/her classmates’ average trait anxiety scores and (2) examined whether this association differed between boys and girls, as well as between elementary school and high school students. During two consecutive school years, 1044 Canadian students (59% girls) from six elementary schools (aged 10–12) and seven high schools (aged 15–17) completed the State-Trait Anxiety Inventory for Children. Multilevel analyses revealed a same-sex peer effect of classmates’ anxiety in girls only (*β* = 0.40, *p* < 0.001). This effect was similar for elementary and high school girls (*β* = 0.07, *p* = 0.27). Interestingly, no association was found for boys, same-sex peers (*β* = 0.11, *p* = 0.25), or opposite-sex peers (*β* = −0.01, *p* = 1.00). Our results suggest that factors related to sex may reinforce anxiety in school settings. Future studies should confirm these results and explore the mechanisms involved in this sex-specific difference.

## 1. Introduction

School is a pivotal learning environment for adolescents, as they spend an average of 923 h per year in their classrooms [1,2]. Yet, adolescents often mention that school is one of their leading sources of anxiety [3,4]. Indeed, situations such as exams, the arrival of a new teacher, or an oral presentation are often perceived as threatening. In turn, this can lead students to experience various levels of anxiety [5,6]. The most widely used framework to study anxiety is the State-Trait Anxiety Model [7,8,9,10]. This model defines state anxiety as a temporary physical (e.g., abdominal discomfort, sweaty hands, rapid heartbeat) and psychological (e.g., worry, nervousness) response to environmental stimuli [9]. According to this model, the level of state anxiety experienced by individuals depends on their level of trait anxiety, while the latter refers to a stable tendency to perceive environmental stimuli as threatening [7,11,12,13,14]. It has been shown that students with high levels of trait anxiety have an enhanced memory of threatening situations [15] and tend to use repetitive thinking. These tendencies are associated with negative mental health outcomes [16,17,18]. As previous studies have reported that even moderate levels of state anxiety can lower the quality of life at school [19,20], affect academic success [21], and increase social difficulties [22,23], it is essential to better understand the factors involved in the experience of state anxiety among students in school.

Peer relationships are particularly important during adolescence (from ages 10 to 19) [24]. Furthermore, adolescents’ behavior tends to be similar to that of their peers [25,26,27,28], a phenomenon known as homophily [29]. As a consequence, recent studies have suggested that peers can play a role in the state anxiety experienced by students. Homophily occurs when youth affiliate with peers with whom they share similarities and engage in the same behaviors over time [28,29]. Researchers have typically conducted multilevel analyses based on longitudinal data to measure the level of shared characteristics between teens and their peers [27,30,31]. As such, homophily has been documented in a variety of adolescent antisocial behaviors (e.g., aggression [32,33,34,35], smoking [36], and drinking, [37,38]) and prosocial behaviors (e.g., helping, sharing, and supporting others [30,39]).in various contexts [25,40,41], including the ties that exist between classmates [28,30]. For example, when academic performance is considered important to students in a classroom, classmates tend to encourage each other to achieve better grades [42]. The same pattern emerges for school engagement, such that students show higher levels of school engagement when surrounded by more engaged classmates [43]. Given that adolescents are prone to imitate their peers in order to feel accepted [24,44], students who are surrounded by classmates with high levels of trait anxiety may display a greater tendency to perceive school-related situations as highly threatening. As a result, this could lead them to experience more state anxiety in school.

The literature exploring the effect of homophily on anxiety remains scarce [45,46,47,48] and has mainly focused on the effect of friends on anxiety. In 2005, a study conducted by Mariano and Harton showed that friends tend to have similar levels of self-reported anxiety symptoms [45]. In 2011, Zalk and colleagues replicated this result in teenagers with social anxiety, which can be characterized by nervousness and discomfort in social situations [48]. They found that friends with social anxiety symptoms can influence each other, such that this can lead to greater individual social anxiety symptoms over time [48]. In 2012, Schwartz-Mette and Rose also tested this effect among friends in a school setting by following a sample of 274 same-sex friend dyads aged 8 to 15 over 6 months [47]. This study also assessed whether students’ sex and age could moderate the effect of friends on a teen’s tendency to experience anxiety, measured via the Children Manifest Anxiety Scale [49]. The results showed that girls experienced anxiety similar to that of their friends, regardless of their age. For boys, this effect was weaker and found only among adolescents [47]. Taken together, studying the effect of friends on anxiety offers insight into the interpersonal dynamics that occur during adolescence. However, it does not provide an accurate representation of what occurs within a classroom environment, as students within the same class are not necessarily friends with one another [2,30]. Yet, even if they are not all friends, students can still influence each other. To further develop the current state of the literature, it is essential to adopt a broader perspective and evaluate the effect of classmates’ trait anxiety on a student’s level of state anxiety.

As shown by previous studies, sex and age are two important variables to consider when examining the effect of peers on various behaviors [25,27,50]. An example of the effect of age is a teen’s tendency to follow the behaviors of their peers, a principle known as susceptibility to peer influence [51,52,53]. Depending on the nature of the behavior, girls and boys respond differently to peer influence. Indeed, girls tend to be sensitive to school-based peer behaviors, whereas boys seem to be sensitive to antisocial behaviors [54]. Given that girls consistently report experiencing more anxiety than boys [55,56,57,58,59,60,61] and that this occurs particularly in school settings, it is of interest to assess whether the effect of classmates’ trait anxiety is different between sexes. It is also important to consider the sex of the classmates in this relationship, as youth are largely influenced by, and interact more often with, same-sex peers [62,63,64]. However, as a greater interest in opposite-sex peers develops during adolescence [24,65,66], youth may also be influenced by opposite-sex peers. To date, no research has explored the influence of opposite-sex peers on anxiety. Therefore, studies should assess the influence of both same-sex and opposite-sex peers on anxiety in youth. Moreover, age seems to influence a teen’s susceptibility to peer influence [27,50,67]. It has been shown that during mid-adolescence (around 15 years of age), youth are more likely to be influenced by their peers than they are during early adolescence (around 10 years of age) [59,60]. Indeed, studies have shown that depending on their age, adolescents are increasingly sensitive to the behaviors of their peers [68,69]. Therefore, it is of interest to evaluate whether the effect of classmates on anxiety is different between elementary (aged 10–12) and high school students (aged 15–16).

Building on previous work using the State-Trait Anxiety Model [7,9], this exploratory study aimed to assess the relationship between a student’s level of state anxiety and their classmates’ level of trait anxiety. This study also explored whether this association is moderated by the sex of the student, sex of the classmates, or age. As this study was exploratory in nature, no hypotheses were formulated.

## 2. Materials and Methods

### 2.1. Study Design

**Figure 1:**
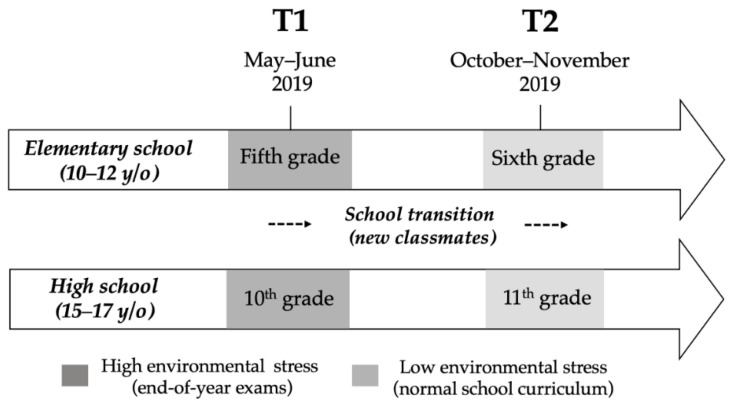
Schematic representation of the original study design conducted by A-A. Journault et al. Elementary school students were followed from Grade 5 (T1) to Grade 6 (T2) and high school students were followed from Grade 10 (T1) to Grade 11 (T2). For both elementary and high school students, T1 occurred at the end of the school year (May and June 2019), which was characterized as a high environmental stress period. T2 occurred at the beginning of the next school year (October and November 2019), during the normal school curriculum, which was characterized as a period of low environmental stress.

The data for this project were derived from a study entitled “My anxiety or your anxiety? Associations between psychological markers of stress and anxiety in children, their parents and teachers”, conducted by the second author of this paper at the Centre for Studies on Human Stress (CSHS). The objective of this doctoral project was to identify the strongest predictor(s) of anxiety experienced by students in schools. Specifically, these factors included students’ individual characteristics, parental factors, and school-related factors. Using multilevel models, this project used a longitudinal research design to examine the effect of classmates on different forms of anxiety in students. During two consecutive school periods in 2019, elementary (10 to 12 years) and high school students (15 to 16 years) completed the State-Trait Anxiety Inventory for Children and other validated questionnaires measuring diverse forms of anxiety in their classrooms. All the measures were collected twice from two different cohorts: an elementary school cohort comprised students who were followed from Grade 5 to Grade 6, and a high school cohort composed of students who were followed from Grade 10 to Grade 11 (see Figure 1). The first measurement time (T1) was at the end of the school year during major exams, which was characterized as a period of high environmental stress for the students. The second measurement time (T2) was at the beginning of the subsequent school year, during a period without major exams, which was characterized as a period of low environmental stress for the students. This methodological design was used to assess whether students’ levels of anxiety vary according to environmental stress.

### 2.2. Participants

The original sample consisted of 1404 students (415 boys, 807 girls) from seven French-speaking elementary schools and six French-speaking high schools in the Montreal area (Quebec, Canada). These schools serve students from various socioeconomic backgrounds. Only students who participated in both measurement times were included in the present analysis in order to compare a student’s level of state anxiety when surrounded by different classmates (classmates were different at T1 and T2). Based on these criteria, 200 students were removed from the analyses. Moreover, an additional 141 students were removed because their class numbers were unidentifiable and, thus, it was impossible to identify their peers. Finally, 19 students from eight classrooms were removed from the analyses because the data pertaining to their classrooms was available for fewer than five of their classmates. These classes were removed because they were not representative of the student’s whole classroom environment (on average, there were 20 students in each class). Taken together, the final sample for this study consisted of 1044 students (415 boys, 619 girls). All the participants were fluent in French. Each participating student was entered into a draw for a chance to win an iPad. One draw was performed per school. Table 1 features the descriptive characteristics of the sample.

### 2.3. Procedure

The study began and ended in 2019 and, thus, did not overlap with the COVID-19 pandemic (which started in March 2020 in Canada). During the 2019 academic year, the students completed self-report questionnaires in their classrooms during a school period of 50 to 75 min under the supervision of the research assistants. During the first measurement time, the details of the questionnaires’ completion varied according to the equipment available in each school. As a result, some students completed the questionnaires using paper and pencil, while others used a secure online platform Studies Web Automation Tool (SWAT) that was developed by the Centre for Studies on Human Stress in Montréal, Canada. At the second measurement time, all the students completed the questionnaires on the online platform SWAT, though one school used paper and pencil due to technical difficulties.

### 2.4. Measures

A demographic questionnaire was administered to all the students to collect information regarding sex, as well as racial and ethnic identity. State and trait anxiety were measured using the French version of the State-Trait Anxiety Inventory for Children (STAI-C) [8,70]. The STAI-C was adapted for children aged 9 to 12 and originates from the State-Trait Anxiety Inventory Revised for Adults (STAI-Y). The French version of this questionnaire was validated by Turgeon and Chartrand (Cronbach’s alpha of 0.89 (state) and 0.88 (trait)) and includes two subscales that measure state anxiety and trait anxiety [70]. Each subscale contains 20 items that can be rated on a 3-point Likert scale. For the subscale measuring state anxiety (STAIC-S), participants are asked about how they feel in the present moment. For each item, they are asked to answer on a scale ranging from “Very [Emotion]” or “[Emotion]” to “Not [Emotion]”. For the subscale measuring trait anxiety (STAIC-T), participants are asked about how they feel in general. For each item, they are asked to answer on a scale ranging from “Hardly ever” or “Sometimes” to “Often”. The scores vary from 20 to 60 for both subscales, in which a high score indicates a greater degree of state/trait anxiety symptoms. Within the current sample, Cronbach’s values were 0.74 for state anxiety and 0.86 for trait anxiety.

### 2.5. Statistical Analyses

The analyses were conducted using R 4.2.1. software of the R Core Team (2021) and the packages lmer4 [71] and multcomp [72]. Two mixed-effects models were employed to determine the effects of time (T1: high environmental stress and T2: low environmental stress) on state anxiety and trait anxiety. To determine potential sex differences in state and trait anxiety, mixed-effect models were selected, as the students (girls and boys) were placed within their classrooms. Initial models were first employed to verify whether the student-level and classroom-level variability in anxiety scores were high enough to justify the use of mixed-effects models. The student’s anxiety score was removed from the average anxiety score of the classroom to avoid capturing the student’s effect on themselves.

To address the primary study objective, linear mixed-effects models (LMM) were conducted to determine the association between the student’s level of state anxiety (dependent variable) and their classmates’ level of trait anxiety (independent variable) in the classroom. Conditional models were then created to examine effect modification by sex or age group. To examine potential sex effects, the classmates’ sex was first included in the model to assess whether a student’s level of state anxiety was predicted more strongly by the average trait anxiety level of the girls or boys in the same class. Next, the sex of the student was entered into the model to assess whether the association was stronger among same-sex peers or opposite-sex peers. Finally, to assess whether the association between the student’s level of state anxiety and their classmates’ level of trait anxiety varied between different age groups, the school level (elementary school vs. high school) was included in the model. The analyses were considered statistically significant at *p* < 0.05.

## 3. Results

### 3.1. Preliminary Analyses

The mean anxiety scores for state anxiety were M_T1_ = 32.4 (*SD* = 6.3) and M_T2_ = 32.6 (*SD* = 6.7). For trait anxiety, the mean anxiety scores for each measurement time were M_T1_ = 37.3 (*SD* = 8.2) and M_T2_ = 37.4 (*SD* = 8.3). Statistically, the anxiety levels did not differ between T1 and T2 for state (*F* (11,017) = 0.21, *p* = 0.26) and trait anxiety (*F* (11,025) = 0.08, *p* = 0.66). Since the anxiety scores did not differ between T1 and T2, the measurement time was not included as a covariate in the subsequent analyses. Significant sex differences were found for the anxiety scores. Girls scored higher for state (*F* (11,043) = 84.6, *p* < 0.001) and trait (*F* (11,079) = 165.9, *p* < 0.001) anxiety than boys. For state anxiety, the mean scores for girls and boys were M_girls_ = 33.7 (*SD* = 0.40) and M_boys_ = 30.6 (*SD* = 0.31). For trait anxiety, the mean scores for girls and boys were M_girls_ = 39.5 (*SD* = 0.38) and M_boys_ = 33.7 (*SD* = 0.36). In addition, the variance of the unconditional model justified the use of two-level hierarchical analyses, given that the intraclass correlations (ICC) were higher than 5% for both state (ICC = 6.71%) and trait anxiety (ICC = 5.0%) [31,73].

### 3.2. Main Analyses

**Main effect of classmates’ level of trait anxiety.** The results of the LMM revealed a statistically significant association between a student’s state anxiety level and the average trait anxiety of the classmates (*β* = 0.36; *p* < 0.001). Individual state anxiety increased by 0.36 units for every increase of one unit in the classroom’s average level of trait anxiety.

**Effect of sex.** When considering only the sex of the classmates, a student’s level of state anxiety was significantly predicted by the trait anxiety of girls in the classroom (*β* = 0.25, *p* < 0.001). However, no significant association was found between a student’s level of state anxiety and the trait anxiety of boys in the classroom (*β* = 0.08, *p* = 0.07). When the sex of the student was added to the model as an interaction term, a same-sex peer effect was found only in girls. Specifically, girls’ state anxiety was significantly associated with the trait anxiety of girls in the same classroom (*β* = 0.40, *p* < 0.001). It was also found that girls’ state anxiety was not significantly associated with boys’ trait anxiety in the same classroom (*β* = 0.14, *p* = 0.12). For boys, there was no opposite-sex peer effect (*β* = −0.01, *p* = 1.00) or same-sex peer effect (*β* = 0.11, *p* = 0.25). Table 2 features the results of the LMMs for the same-sex and opposite-sex peer interaction effects.

**Effect of school level.** The associations between a student’s level of state anxiety and the trait anxiety level of their classmates did not vary between school levels (*β* = 0.05, *p* = 0.60). Table 3 features the results of this model.

## 4. Discussion

The goal of the current study was to assess whether an association exists between a student’s level of state anxiety and their classmates’ trait anxiety and, if so, to evaluate whether this association differs according to the sex and age of the students. The findings of the current study suggest that a student’s state anxiety is associated with their classmates’ trait anxiety. Furthermore, this association is more prevalent in girls than in boys but is independent of the school-age level.

As previous studies have focused solely on the effect of friends on anxiety [45,46,47,48], no study to date has examined the effect of classmates on the state anxiety experienced by teenagers in school. Thus, this study provides a unique contribution to the literature, as youth in classroom settings are exposed to the behaviors (and anxiety) of not only their friends but also each of their classmates [2,32]. This finding aligns with those of previous literature stating that classmates play an important role in an adolescent’s life [2,30] and their ability to impact a student’s state anxiety. To better understand the nature of this association, future research should consider each individual student’s anxiety levels to determine how this affects his/her reactivity to to classmates’ anxiety. For instance, compared to individuals who are already highly anxious, students who are initially less anxious may react more strongly to the trait anxiety of their classmates.

The results of this study revealed that girls and boys respond differently to the trait anxiety of their classmates. Specifically, girls appeared to be sensitive to the trait anxiety of same-sex peers, whereas no association was found in boys for either same or opposite-sex peers. Given that both girls and boys spend a vast majority of their time with same-sex peers [24,62,64], it is unclear why a homophilic effect of anxiety was found only in girls. Several hypotheses could explain this result. For example, girls may send a greater number of anxiety signals to their classmates than boys. In turn, this could increase the probability that same-sex classmates (girls) express similar levels of anxiety. A well-recognized finding in the literature is that girls are socialized to express a variety of emotions at a greater level than boys [74,75], including anxiety [55,56,57,58,60,61]. The results of the present study showed that compared to boys, girls scored higher on the state and trait anxiety scales. Girls may express greater anxiety than boys due to gendered social norms in the classroom, where these norms are informed by implicit and explicit rules about how each sex is supposed to behave [76,77]. In consequence, this could mean that it is more acceptable for girls to express feelings of anxiety [76,78], whereas emotions could be seen as a sign of vulnerability and inferiority in boys [74]. In a similar vein, it is common for adolescents to adopt behaviors that conform to these social norms [25], as it guarantees their social acceptance [77]. This specific hypothesis could be tested in future studies by assessing gender social norms in the classroom. Overall, this finding suggests that factors related to sex may reinforce anxiety in school settings.

No significant effect of age was found, such that the association between a student’s state anxiety and classmates’ trait anxiety level was similar among elementary (ages 10–12) and high school girls (ages 15–16). Based on this result, it seems that students respond similarly to the trait anxiety of their classmates, regardless of their age. It is difficult to contextualize this finding, given the inconsistency of the literature. For example, several studies have found an effect of age, such that 15-year-old adolescents were more likely to be influenced by their peers than younger teenagers (aged 10) [25,50,67]. That said, the latter findings have been criticized, as they focus exclusively on antisocial behaviors. Moreover, a recent meta-analysis examining the effects of peers on various behaviors (including anxiety) found no effect of age [27]. Taken together, the moderating role of age on different types of behavior (including anxiety) is currently inconclusive in this domain. As a result, longitudinal studies following individuals throughout adolescence are required to clarify how youth respond to the behaviors of their peers as they age.

This study was the first to explore the effect of classmates on students’ state anxiety. The main strengths of this study were the large sample size, as well as the consideration of same-sex and opposite-sex peer effects. This study provides a better understanding of the role of classmates in the state anxiety experienced by students in school. By underscoring the fact that girls and boys respond differently to the trait anxiety of their peers, this study also suggests that it is important to consider the sex of the individuals and of the classmates as moderating variables in future studies. Overall, this is a fruitful research avenue, as it raises awareness about the impact of classmates on one’s anxiety. Beyond this, it could eventually contribute to the development of strategies that aim to promote students’ well-being despite being surrounded by anxious peers.

This study had certain limitations. First, the study was correlational, which precludes any statements of causality or directionality. The results of the study only allow us to conclude that an association exists between a girl’s level of state anxiety and the average trait anxiety of other girls in her classroom. Studies based on experimental research designs (e.g., social network analysis) should be conducted to identify the individuals with whom each student interacts most frequently [28]. Social network analysis creates a schematic representation of the social relationships among youth, allowing for a more complete understanding of the complex relationships between youth. Secondly, data were not collected from all the students within a class, which resulted in a loss of data from classes with fewer students. This may have resulted in selection bias and may have reduced the external validity of the current findings. To gain a complete understanding of the classroom dynamic, future studies should collect data from every student in the class. Thirdly, the current findings may not be generalized to other forms of anxiety, such as test anxiety or anxiety sensitivity. Furthermore, as the sample was composed of youth and teens of a similar socioeconomic status and culture, the current findings may not be generalized to youth from more diverse cultures and socioeconomic backgrounds. Finally, another limitation of the study is that the homophily effect was analyzed during two measurement times that represented different periods of the school year. Given that familiarity promotes peer influence [28,29], peers may have been more influential at the first measurement time (end of the school year), as the students had time to become acquainted with one other and create friendships, whereas at the second measurement time (beginning of the school year), the students had only just met one another. To control for this variable and to determine whether school periods affect homophily, future studies should measure and compare the degree of familiarity among classmates at the beginning and end of the school year.

## 5. Conclusions

The current exploratory study assessed the relationship between a student’s state anxiety level and the average trait anxiety level of his/her classmates. It was found that a girl’s state anxiety is significantly associated with the level of trait anxiety of the girls in the same classroom, an effect that was not observed in boys. These results suggest a potential sex-dependent homophilic effect of anxiety in schools. Further research is required to confirm these results in other school settings.

## Figures and Tables

**Table 1:** Descriptive characteristics of the final sample.

	Cohorts		Total
	Elementary Schools	High Schools	
Schools	7	6	13
Classrooms ^1^	17	33	50
Participants	266	778	1044
Male	116	299	415
Female	143	476	619
Age	10–12	15–17	-
Racial and ethnic identity ^2^			
White	140	145	285
Indigenous	2	2	4
Middle Eastern	1	1	2
Asian	1	2	3
Black	2	0	2
Central/Southern American	4	4	8
Other	44	20	64
Missing	88	588	676

Note: Descriptive characteristics of the final sample (schools, classrooms, sex, age, racial and ethnic identity) by cohort. ^1^ Number of participants per classroom ranged from 5 to 34. ^2^ Data regarding the origins of the student were collected through a demographic questionnaire answered by the parent.

**Table 2:** Results of the linear mixed-effects models for the same-sex and opposite-sex peer interaction effects.

	Estimate	SE	Z Value	*p* (>|z|)
Effect of girls on girls	0.40	0.05	7.47	<0.001
Effect of girls on boys	0.14	0.07	2.15	0.12
Effect of boys on girls	−0.01	0.04	−0.15	1.00
Effect of boys on boys	0.11	0.06	1.89	0.25

**Table 3:** Linear mixed-effects models of the association between a student’s level of state anxiety and their classmates’ level of trait anxiety, as well as the effect of the school level.

Models	Effects	Estimate	SE	df	t	*p* (>|t|)
1	Student’s level of state anxiety (intercept)	19.22	1.95	149	9.87	<0.001
	Classmates’ trait anxiety	0.36	0.05	147	6.86	<0.001
2	Elementary school students’ level of state anxiety (intercept)	21.71	3.0	1387	7.30	<0.001
	High school students’ level of state anxiety	0.03	0.30	1798	0.06	>0.94
	β for school level	0.05	0.10	1753	0.53	>0.60

## Data Availability

The current study was pre-registered on the OSF platform on 9 December 2021 (https://osf.io/vdkue/, accessed on 8 November 2022). The anonymized data and the R code used for the analyses were also deposited (https://osf.io/cr8xt/, accessed on 8 November 2022). As planned in the pre-registration, we conducted separate multilevel models assessing trait anxiety and state anxiety and two other forms of anxiety (test anxiety and anxiety sensitivity). As the results were significant for each form of anxiety, we wrote the article on state anxiety and trait anxiety as per the most widely used framework in the anxiety field: the State-Trait Anxiety Model. This allowed us to explain the results in a straightforward manner and control for differences between constructs measured by different questionnaires. Moreover, descriptions of all the questionnaires used in the larger study conducted by A.A.-J. are available at https://osf.io/4jdvw/, accessed on 8 November 2022).
